# Therapeutic effect and rebound evaluation of dapagliflozin on glycated hemoglobin (HbA1c) in type 1 diabetes mellitus patients

**DOI:** 10.3389/fphar.2022.972878

**Published:** 2023-01-04

**Authors:** Dong-Dong Wang, Cun Zhang, Ke Hu, Su-Mei He, Ping Zhu, Xiao Chen

**Affiliations:** ^1^ Jiangsu Key Laboratory of New Drug Research and Clinical Pharmacy and School of Pharmacy, Xuzhou Medical University, Xuzhou, China; ^2^ Department of Pharmacy, Xuzhou Oriental Hospital Affiliated to Xuzhou Medical University, Xuzhou, China; ^3^ Department of Pharmacy, Suzhou Science and Technology Town Hospital, Suzhou, China; ^4^ Department of Endocrinology, Huaian Hospital of Huaian City, Huaian, China; ^5^ School of Nursing, Xuzhou Medical University, Xuzhou, China

**Keywords:** therapeutic effect, rebound evaluation, dapagliflozin, HbA1c, type 1 diabetes mellitus

## Abstract

Dapagliflozin has been used to treat patients with type 1 diabetes mellitus; however, the actual drug efficacy of dapagliflozin on glycated hemoglobin (HbA1c) and whether there is a rebound from dapagliflozin efficacy on HbA1c remain unknown. The present study aimed to explore the actual therapeutic effect and rebound situation of dapagliflozin on HbA1c in type 1 diabetes mellitus patients. A total of 1,594 type 1 diabetes mellitus patients were enrolled for analysis using a non-linear mixed effect model from randomized controlled trials from published literature works including two 5 mg/day dapagliflozin dosage groups and three 10 mg/day dapagliflozin dosage groups. The change rate of HbA1c from a baseline value was chosen as a dapagliflozin pharmacodynamic evaluation index. After deducting control group effects, the therapeutic effect of 5 and 10 mg/day dapagliflozin on HbA1c in type 1 diabetes mellitus patients had no significant difference. In addition, the actual maximal efficacy (AE_max_) of dapagliflozin on HbA1c was -6.24% at week 9. When it reached the AE_max_, the dapagliflozin pharmacodynamic rebound on HbA1c occurred, and when the treatment was continued for 0.5 and 1 year, the actual efficacies were -4.70% (75% AE_max_) and -3.27% (52% AE_max_), respectively. This was the first time to clarify the actual therapeutic effect and rebound situation of dapagliflozin on HbA1c in type 1 diabetes mellitus patients, providing a reference value for clinical practices.

## 1 Highlight

After deducting control group effects, the therapeutic effect of 5 and 10 mg/day dapagliflozin on HbA1c in T1DM patients had no significant difference.
The actual maximal efficacy (AE_max_) of dapagliflozin on HbA1c was -6.24% at week 9. When it reached the AE_max_, the dapagliflozin pharmacodynamic rebound on HbA1c occurred, and when the treatment was continued for 0.5 and 1 year, the actual efficacies were -4.70% (75% AE_max_) and -3.27% (52% AE_max_), respectively.
This was the first time to clarify the actual therapeutic effect and rebound situation of dapagliflozin on HbA1c in T1DM patients, providing a reference value for clinical practices.

## 2 Introduction

Diabetes mellitus (DM) is a group of metabolic diseases characterized by hyperglycemia, which is caused by defective insulin secretion, impaired insulin biology, or both. Long-term hyperglycemia leads to chronic damage and dysfunction of various tissues, especially eyes (Yao et al., 2022), kidneys (Georgianos et al., 2022), heart (Qiu et al., 2022), blood vessels (Li et al., 2016), and nerves (Mendez-Morales et al., 2022). In 2010, the estimated prevalence of DM in adults worldwide was 6.4%, and it will probably rise up to 7.7% by 2030 (Shaw et al., 2010; Margaritis et al., 2021). Type 1 diabetes mellitus (T1DM), encompassing 5–10% of the total DM cases, is an autoimmune disease characterized by the destruction of pancreatic β-cells (Wallberg and Cooke, 2013), whose development relies on at least three key factors: genetic predisposition, environmental aspect, and immunoreaction that leads to β-cell destruction (Esposito et al., 2019; Purdel et al., 2021).

At present, T1DM is regulated using strict blood sugar control, exogenous insulin supplementation, dietary adjustment, appropriate exercise, and other physiological factors (Dikeman and Westman, 2021), among which insulin-replacement treatment is a cornerstone therapy for T1DM patients (American Diabetes Association, 2019). With the progress in insulin delivery and glucose monitoring systems, the treatment environment for T1DM patients has been significantly improved; however, the blood glucose control of T1DM patients is still not ideal, and only less than 1/3 patients can achieve the ideal blood glucose control (Miller et al., 2015; Paik and Blair, 2019). Therefore, it is urgent to find a new treatment for T1DM patients with poor glycemic control.

Dapagliflozin, a sodium–glucose cotransporter-2 inhibitor, has been approved for the treatment of type 2 DM (T2DM) (Curovic et al., 2022; Lazzaroni et al., 2022). The mechanism of dapagliflozin is to promote glycemic disposal in an insulin-independent way, thereby decreasing postprandial glycemia and glycemic excursions accompanied by an inferior insulin demand without increasing the risk of hypoglycemia (Li et al., 2022). It is interesting to note that research results suggest that dapagliflozin is a promising adjunct treatment to insulin which improves glycemic control in patients with inadequately controlled T1DM (Dandona et al., 2017; Mathieu et al., 2018; Parkinson et al., 2019; Araki et al., 2020; Araki et al., 2021; Biester et al., 2021); meanwhile, it significantly decreases the glycated hemoglobin (HbA1c) (Li et al., 2022). HbA1c identifies average plasma glucose concentration, and for people with diabetes this is important as the higher the HbA1c, the greater the risk of developing diabetes-related complications (Rydberg et al., 2022; Wikstrom et al., 2022). However, the actual drug efficacy of dapagliflozin on HbA1c and whether there is a rebound from dapagliflozin efficacy on HbA1c remain unknown and thus cannot effectively guide an appropriate use of clinical medication. The present study aimed to explore the actual therapeutic effect and rebound situation of dapagliflozin on HbA1c in T1DM patients.

## 3 Methods

### 3.1 Included patients

A total of 1,594 T1DM patients treated with dapagliflozin were enrolled from published literature works, which were randomized controlled trials (Kuhadiya et al., 2016; Dandona et al., 2018; Mathieu et al., 2020), and they were collected for analysis using a non-linear mixed effect model. These research studies were approved by the ethics committee of each participating center (Kuhadiya et al., 2016; Dandona et al., 2018; Mathieu et al., 2020). In the present study, information on source, group, dapagliflozin dosage, duration of treatment, HbA1c, number of people, age, duration of T1DM, and total baseline insulin dose was collected from these randomized controlled trials.

The change rate of HbA1c from the baseline value was chosen as a dapagliflozin pharmacodynamic evaluation index in order to eliminate the potential baseline effect, and it is given in Equation 1 as follows:

$$H\%=\frac{H_{t}-H_{b}}{H_{b}}\times 100\%$$ (1)

where H_t_ represents the value of HbA1c at time and H_b_ represents the value of HbA1c at baseline.

### 3.2 Model establishment

To obtain the actual dapagliflozin effect on HbA1c in T1DM patients, the control effect needs to be subtracted from the sum effect, which is shown in Eq. 2:

$$H_{actuali,j}=H_{sumi,j}–H_{control,i,j}$$ (2)

Here, H_sumi,j_ represents the sum effect of dapagliflozin on HbA1c in T1DM patients; H_control,i,j_ represents the control group effect on HbA1c in T1DM patients; H_actual,i,j_ represents the actual effect of dapagliflozin on HbA1c in T1DM patients; i represents different studies; and j represents the timepoint of every study.

We analyzed actual dapagliflozin effect on HbA1c in T1DM patients and found that it increased over time; at some timepoint, a maximal value was reached, followed by a drug rebound effect, which is shown in the Supplementary Data. These data characteristics were described using the following Eq. 3:

$$E_{actual,i,j}=\frac{E_{\max,\;i,\;j\;}\times Time}{{ET}_{50,\;i,\;j\;}+Time}{\times e}^{\tau_{i,j}\times Time}+\frac{{Ɛ}_{i,\;j}}{\sqrt{\frac{N_{i,\;j}}{100}}}$$ (3)

Here, E_actual,i,j_ represents the actual effect of dapagliflozin on HbA1c in T1DM patients; E_max_ represents the theoretical maximal effect of dapagliflozin on HbA1c in T1DM patients; ET_50_ represents the treatment duration to reach half of the E_max_; τ is the rate of drug rebound; i represents different studies; and j represented the timepoint of every study. Ɛ _i,j_ represents the residual error of the study i with j time and N_i, j_ represents the sample size in the study i with timepoint j. Ɛ_i,j_ was weighted by the sample size, assumed to be normally distributed, with a mean of 0 and variance of σ^2^/(N_i,j_/100).

The additive or exponential error models were used to assess the inter-study variabilities, which were shown in Eqs 4–9as follows:

$$E_{\max,i,j}=E_{\max}+\eta_{1,i}$$ (4)

$${ET}_{50,i,j}={ET}_{50}+\eta_{2,i}$$ (5)

$$\tau_{i,j}=\tau +\eta_{3,i}$$ (6)

$$E_{\max,i,j}=E_{\max}\times \exp\left(\eta_{1,i}\right)$$ (7)

$${ET}_{50,i,j}={ET}_{50}\times \exp\left(\eta_{2,i}\right)$$ (8)

$$\tau_{\;i,j}=\tau \times \exp\left(\eta_{3,i}\right)$$ (9)

Here, η_1,i_, η_2,i_, and η_3,i_ represent the inter-study variabilities, and when available, they would be incorporated into E_max_, ET_50_, or τ, respectively. η_1,i_, η_2,i_, and η_3,i_ were assumed to be normally distributed, with a mean of 0 and variance of ω_1,i_^2^, ω_2,i_^2^, and ω_3,i_^2^, respectively.

The categorical covariates or continuous covariates were evaluated using Eqs 10–12 :

$$H_{p}=H_{T}+COV\times \theta_{C}$$ (10)

$$H_{p}=H_{T}+\left(COV-{COV}_{m}\right)\theta_{c}$$ (11)

$$H_{p}=H_{T}\times {\left(COV/{COV}_{m}\right)}^{\theta c}$$ (12)

Here, H_p_ represents the parameter for a patient with a covariate value of COV; H_T_ represents the typical value of the parameter; COV represents covariate; COV_m_ represents the median value of covariables in the population; and θ_c_ represents a correction coefficient of the covariate to the model parameter.

The model was built up *via* non-linear mixed effect modeling (NONMEM, edition 7, ICON Development Solutions, Ellicott City, MD, United States of America) software. After the basic model was built, when available, potential covariates were considered to be incorporated into E_max_, ET_50_, or τ. The change in the objective function value (OFV) was the covariate inclusion criteria, where if the OFV decrease was greater than 3.84 (χ^2^, α *=* 0.05, d.f. = 1), it was considered sufficient as inclusion and if the OFV increase was greater than 6.63 (χ^2^, α *=* 0.01, d.f. = 1), it was considered sufficient as significance in the final model (Wang et al., 2021).

### 3.3 Model evaluation

The goodness-of-fit plots of the model (observations vs*.* predictions and observations/predictions vs*.* time), individual plots, and conditional weighted residual (CWRES) vs*.* time/population predictions/individual predictions were used to estimate the final model, where CWRES values in the range of -3 to 3 represented a good prediction of the model (Chen et al., 2021; Chen et al., 2022). In addition, the medians and 2.5–97.5% results from bootstrap (*n* = 1,000) were used to compare with final model parameters. Prediction-corrected visual predictive check (VPC) plots were used to assess the predictive performance of the final model.

### 3.4 Model prediction

The actual efficacy curve of dapagliflozin on HbA1c from the final model was simulated using the Monte Carlo method, which was a mathematical technique used to predict the situation in which an event would occur (Wang et al., 2021; Wang et al., 2022). The actual maximal efficacy (AE_max_) of dapagliflozin on HbA1c and the dapagliflozin therapeutic effect on HbA1c after reaching AE_max_ when continued for 0.5 and 1 year were simulated.

## 4 Results

### 4.1 Included studies

A total of 1,594 multinational T1DM patients were enrolled for analysis from three randomized controlled trials from published literature works including two 5 mg/day dapagliflozin dosage groups and three 10 mg/day dapagliflozin dosage groups (Kuhadiya et al., 2016; Dandona et al., 2018; Mathieu et al., 2020). In addition, the duration of dapagliflozin treatment was from 12 to 52 weeks, the age of T1DM patients were in the range of 41.9–55.0 years, the baseline value of HbA1c ranged from 7.40 to 8.53%, the duration of T1DM ranged from 18.98 to 30 years, and the total baseline insulin dose was in the range of 44.7–63.1 IU, which are shown in the Supplementary Data. Part of the literature information was retrieved from the previous study (Wang et al., 2022).

### 4.2 Modeling

In the final model, no covariate (in particular dosage) was incorporated into the model, showing no significant dose–response relationship between 5 and 10 mg/day affecting HbA1c, according to the current research studies. The theoretical E_max_ and ET_50_ from dapagliflozin effects on HbA1c were -8.08% and 1.23 weeks, and the rate of drug rebound, τ, was -0.0145 weeks^−1^. The actual therapeutic effect of dapagliflozin on HbA1c in T1DM patients was shown in Eq. 13as follows:

$$E_{actual}=\frac{-8.08\%\times Time}{\;1.23+Time}{\times e}^{-0.0145\times Time}$$ (13)

Here, E_actual_ represents the actual therapeutic effect of dapagliflozin on HbA1c in T1DM patients and Time represents a variable for dapagliflozin treatment duration in T1DM patients.

### 4.3 Evaluation

**FIGURE 1:**
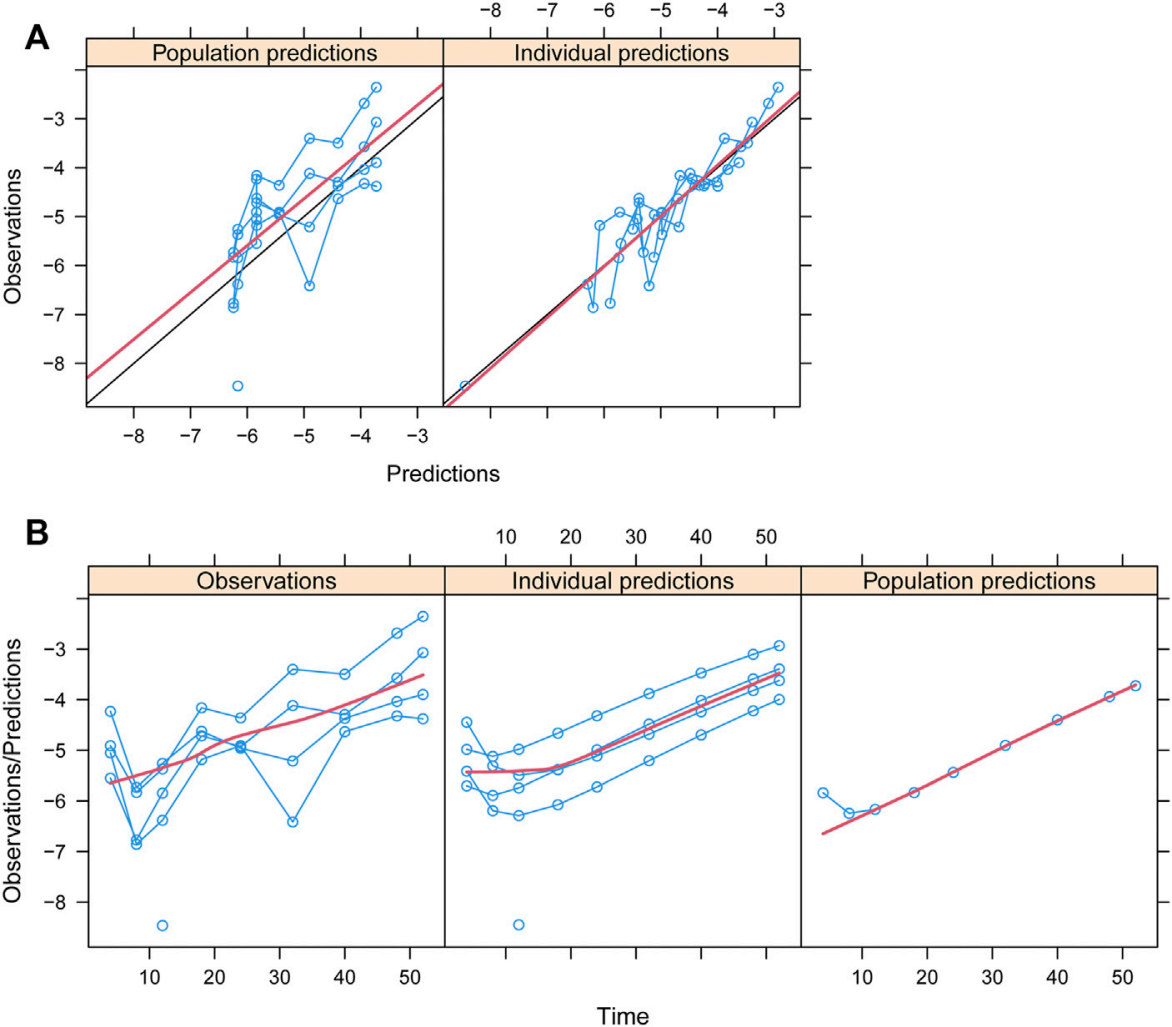
Goodness-of-fit plots of the model. **(A)** Observations vs. predictions and **(B)** observations/predictions vs. time. Blue smooth lines represent the trend of an individual study. Red smooth lines represent the trend of all points.

**FIGURE 2:**
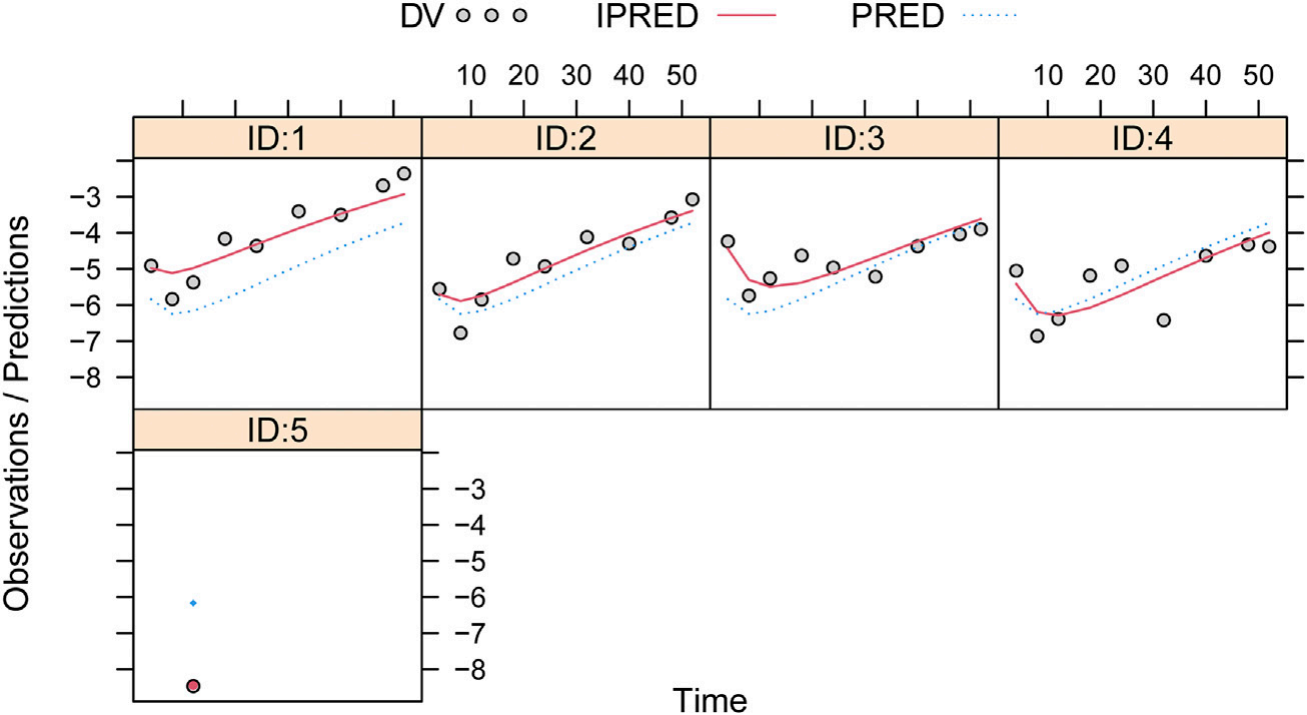
Individual plots. IDs:1–5 were five dapagliflozin dosage groups, of which two of them were 5 mg/day and three of them were 10 mg/day (Kuhadiya et al., 2016; Dandona et al., 2018; Mathieu et al., 2020).

**FIGURE 3:**
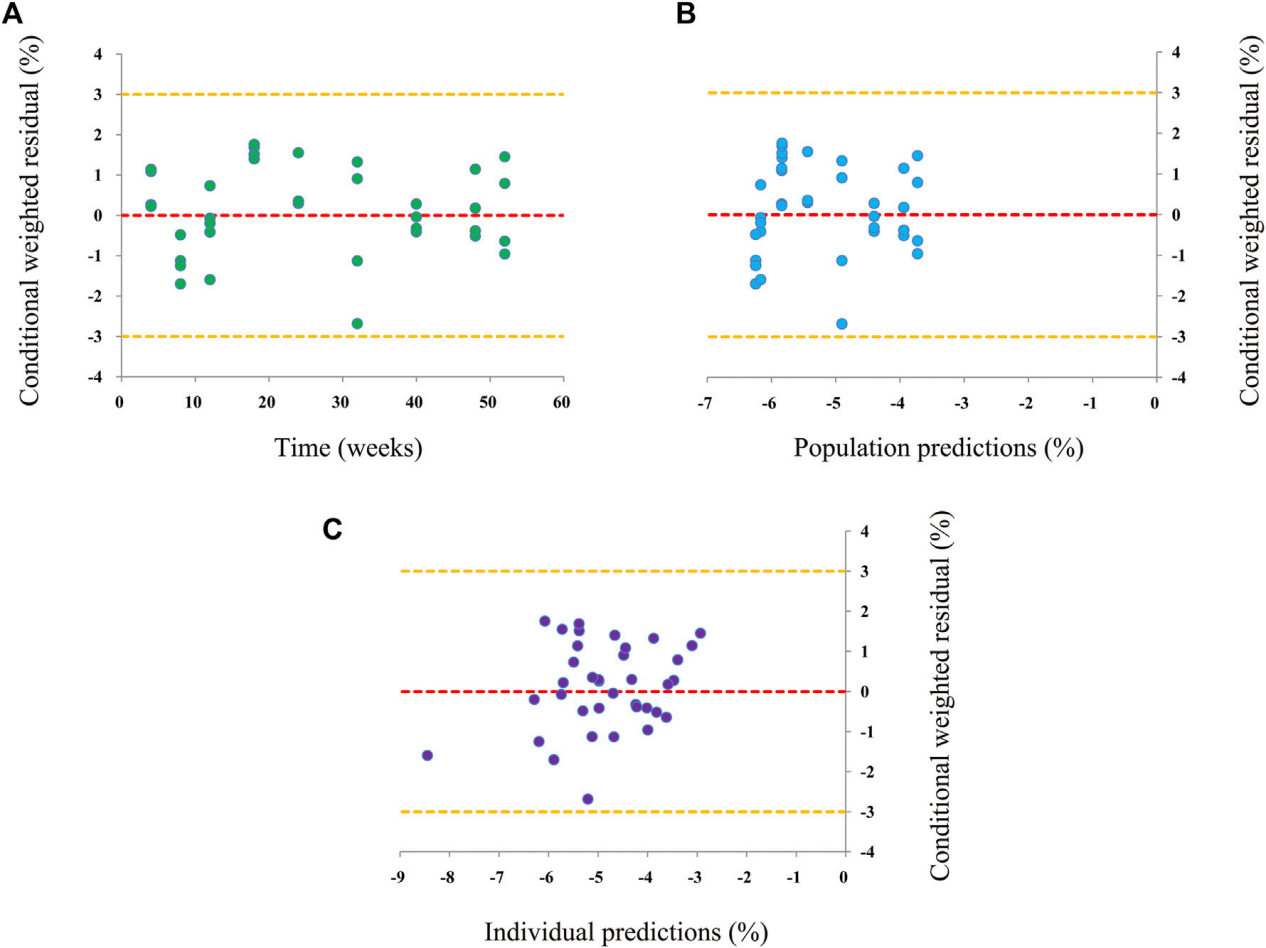
Conditional weighted residual of the final model. **(A)** Conditional weighted residual (CWRES) vs*.* time, **(B)** CWRES vs*.* population predictions, and **(C)** CWRES vs*.* individual predictions.

**FIGURE 4:**
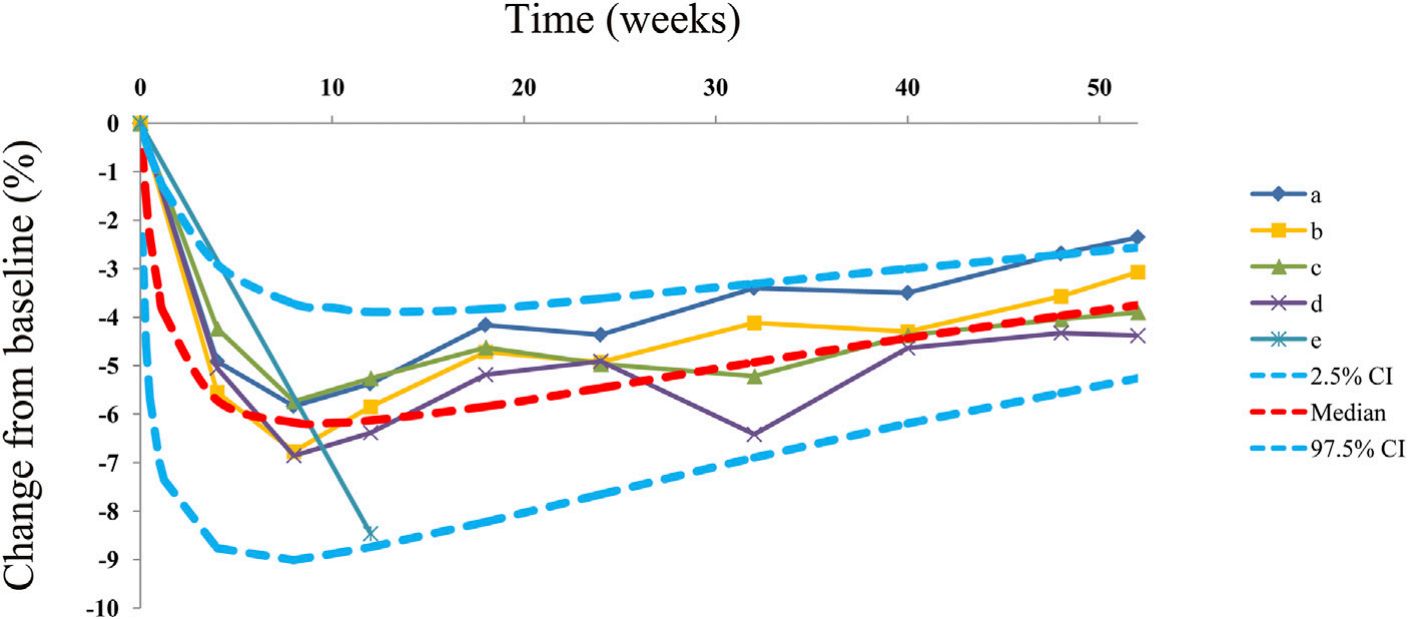
Prediction-corrected visual predictive check plots. Median, 2.5% CI, and 97.5% CI were simulated using the Monte Carlo method (*n* = 1,000); CI, confidence interval. (a-e) Five dapagliflozin dosage groups from three randomized controlled trials (Kuhadiya et al., 2016; Dandona et al., 2018; Mathieu et al., 2020).

Observations vs*.* predictions and observations/predictions vs*.* time are shown in Figures 1A, B, respectively, showing good linear relationships between predictions and observations. Individual plots are shown in Figure 2, indicating that the present model could well fit the situation of each dose group. CWRES vs*.* time/population and predictions/individual predictions are shown in Figure 3, and the CWRES values were all ranged between -3 and 3, indicating the better fitting of the final model. Furthermore, the medians and 2.5–97.5% results from bootstrap are shown in Table 1. Medians from bootstrap were equal to or approximately equal to the corresponding estimate values of the final model, and all the bias absolute values were less than 15%, indicating that the model was stable. Prediction-corrected VPC plots are shown in Figure 4 and the most observed data were included in the 95% prediction intervals produced with simulation data, indicating the better predictive power of the final model.

**TABLE 1:** Parameter estimates of the final model and 95% confidence interval.

Parameter	Estimate	Simulation		Bias (%)
		Median	95% confidence interval	
E_max_, %	-8.08	-8.10	[-9.16, -7.24]	0.248
ET_50_, week	1.23	1.23	[0.88, 1.69]	0
*τ*, week^−1^	-0.0145	-0.0145	[-0.0171, -0.0126]	0
ω_Emax_	0.189	0.187	[0.099, 0.220]	-1.058
ω_ET50_	0.742	0.641	[0.242, 1.015]	-13.612
Ɛ	0.301	0.298	[0.237, 0.321]	-0.997

95% confidence interval is shown with 2.5th and 97.5th percentiles; E_max_ represents the theoretical maximal effect; ET_50_ represents the treatment duration to reach half of E_max_; τ represents the rate of drug rebound; ω_Emax_ represents the inter-study variability of E_max_; ω_ET50_ represents the inter-study variability of ET_50_; Ɛ represents the residual error; and Bias = (Median-Estimate)/Estimate × 100%.

### 4.4 Prediction

**FIGURE 5:**
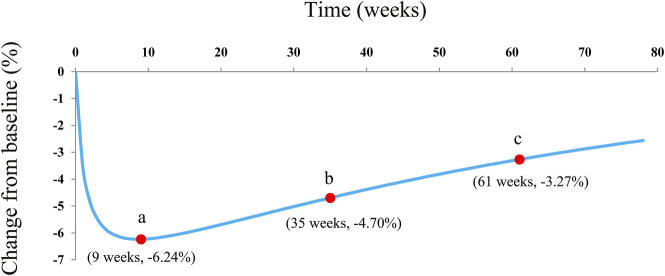
Model prediction. **(A)** Actual maximal efficacy (AE_max_) of dapagliflozin on HbA1c; **(B)** after it reached the AE_max_, the dapagliflozin therapeutic effect on HbA1c continuing the treatment for 0.5 years (26 weeks), at week 35; and **(C)** after it reached the AE_max_, the dapagliflozin therapeutic effect on HbA1c continuing the treatment for 1 year (52 weeks) at week 61.

The actual efficacy curve of dapagliflozin on HbA1c from the final model is shown in Figure 5, and the AE_max_ of dapagliflozin on HbA1c was -6.24% at week 9. Meanwhile, it was found that the AE_max_ was less than the theoretical E_max_ (-8.08%). This phenomenon was mainly due to the dapagliflozin pharmacodynamic rebound existing on HbA1c. After it reached the AE_max_, the actual efficacies of dapagliflozin on HbA1c when continued for 0.5 and 1 year were -4.70% (75% AE_max_) and -3.27% (52% AE_max_), respectively.

## 5 Discussion

Dapagliflozin is the first approved sodium–glucose cotransporter-2 inhibitor for the treatment of T2DM, which can be regarded as an important choice in diabetes drug therapy and can be used as an adjunct to diet and exercise to improve blood glucose control in adults with T2DM. A growing body of research studies now suggests that the benefits of dapagliflozin go beyond that. It was reported that dapagliflozin had potential therapeutic efficacy for non-alcoholic fatty liver disease, including significantly decreasing hepatic enzymes and metabolic indicators and improving body compositions (He et al., 2022). In addition, dapagliflozin represented a new pharmacologic option for reducing chronic kidney disease progression in patients with and without diabetes (Kelly, 2022). In addition these, dapagliflozin administration in T2DM patients resulted in both acute and chronic reduction in systolic blood pressure, a reduction in vasoconstrictors, and an increase in vasodilators (Ghanim et al., 2021). These changes may potentially contribute to its antihypertensive effects and its benefits in congestive cardiac failure (Ghanim et al., 2021; Hodrea et al., 2022).

Moreover, Melin et al. (2022) found that dapagliflozin pharmacokinetics was similar in adults with T1DM and T2DM. In addition, it was interesting to note that more and more research results suggested that dapagliflozin was a promising adjunct treatment to insulin which improves glycemic control in patients with inadequately controlled T1DM (Dandona et al., 2017; Mathieu et al., 2018; Parkinson et al., 2019; Araki et al., 2020; Araki et al., 2021; Biester et al., 2021). Wang et al. (2022) explored the quantitative relationship between dapagliflozin and loss of weight in T1DM patients and found that to achieve the plateau period in the loss of weight, 5 mg/day dapagliflozin was required for at least 41.6 weeks. Li et al. (2022) found that dapagliflozin treatment significantly decreased HbA1c, insulin dosage, and body weight without increasing the risk of hypoglycemia in T1DM. However, the actual drug efficacy of dapagliflozin on HbA1c and whether there is a rebound from dapagliflozin efficacy on HbA1c remained unknown and thus cannot effectively guide an appropriate use of clinical medication. The present study aimed to explore the actual therapeutic effect and rebound situation of dapagliflozin on HbA1c in T1DM patients.

In the present study, a total of 1,594 T1DM patients were enrolled for analysis from three randomized controlled trials from published literature works including two 5 mg/day dapagliflozin dosage groups and three 10 mg/day dapagliflozin dosage groups (Kuhadiya et al., 2016; Dandona et al., 2018; Mathieu et al., 2020). Parkinson et al. (2019) reported that baseline HbA1c was found to have an impact on HbA1c reductions when treated with dapagliflozin; that is, patients with higher HbA1c baseline were predicted to have greater HbA1c reductions than those with lower baseline levels. In order to eliminate the potential baseline effect, the change rate of HbA1c from the baseline value was chosen as the dapagliflozin pharmacodynamic evaluation index in the present study. In addition, to obtain the actual dapagliflozin effect on HbA1c in T1DM patients, the control effect was subtracted from the sum effect.

In the final model, no covariate (in particular dosage) was incorporated into the model, showing no significant dose–response relationship between 5 and 10 mg/day dapagliflozin affecting HbA1c, according to the current research studies. The theoretical E_max_ and ET_50_ from dapagliflozin effects on HbA1c were -8.08% and 1.23 weeks, and the rate of drug rebound, τ, was -0.0145 weeks^−1^. In addition, the overall evaluation of the model of dapagliflozin on HbA1c in T1DM patients was good. As for the current doses, 5 and 10 mg/day dapagliflozin had no significant dose–response relationship on HbA1c, which was similar to previous research studies that showed no significant dose–response relationship between 5 and 10 mg/day dapagliflozin affecting the loss of weight in T1DM patients (Wang et al., 2022). Subsequently, the present study simulated the actual efficacy curve of dapagliflozin on HbA1c from the final model, and the AE_max_ of dapagliflozin on HbA1c was -6.24% at week 9. Meanwhile, it was found that the AE_max_ was less than the theoretical E_max_ (-8.08%), which was mainly due to the dapagliflozin pharmacodynamic rebound existing on HbA1c. After it reached the AE_max_, the actual efficacies of dapagliflozin on HbA1c when the treatment was continued for 0.5 and 1 year were -4.70% (75% AE_max_) and -3.27% (52% AE_max_), respectively. In other words, the AE_max_ of dapagliflozin on HbA1c appeared at week 9 of continuous treatment with dapagliflozin, and at week 35 and week 61, the drug effects were approximately equal to three-quarters AE_max_ and half AE_max_, respectively. In particular, this kind of pharmacodynamic rebound is not uncommon. Dong et al. (2017) reported five long-term weight loss drugs that also had a rebound phenomenon. However, the underlying mechanism of the existence of a rebound from dapagliflozin efficacy on HbA1c remains unknown, and explorations are needed in future research.

Of course, this study also has some limitations. For example, at present, there are few finished clinical studies on dapagliflozin therapeutic effect on patients with T1DM, and the number of studies currently included is limited and needs to be expanded in the future.

## 6 Conclusion

This was the first time to clarify the actual therapeutic effect and rebound situation of dapagliflozin on HbA1c in T1DM patients, providing a reference value for clinical practices.

## Data Availability

The original contributions presented in the study are included in the article/Supplementary Material; further inquiries can be directed to the corresponding authors.
